# Association between perioperative plasma transfusion and in-hospital mortality in patients undergoing surgeries without massive transfusion: A nationwide retrospective cohort study

**DOI:** 10.3389/fmed.2023.1130359

**Published:** 2023-02-15

**Authors:** Xiaohan Xu, Yuelun Zhang, Bo Tang, Xuerong Yu, Yuguang Huang

**Affiliations:** ^1^Department of Anesthesiology, Chinese Academy of Medical Sciences & Peking Union Medical College Hospital, Beijing, China; ^2^Medical Research Center, Chinese Academy of Medical Sciences & Peking Union Medical College Hospital, Beijing, China

**Keywords:** fresh frozen plasma, in-hospital mortality, non-massive transfusion, real-word data, nationwide, surgical patients

## Abstract

**Background:**

An aggressive plasma transfusion is associated with a decreased mortality in traumatic patients requiring massive transfusion (MT). However, it is controversial whether non-traumatic or non-massively transfused patients can benefit from high doses of plasma.

**Methods:**

We performed a nationwide retrospective cohort study using data from Hospital Quality Monitoring System, which collected anonymized inpatient medical records from 31 provinces in mainland China. We included the patients who had at least one record of surgical procedure and received red blood cell transfusion on the day of surgery from 2016 to 2018. We excluded those receiving MT or diagnosed with coagulopathy at admission. The exposure variable was the total volume of fresh frozen plasma (FFP) transfused, and the primary outcome was in-hospital mortality. The relationship between them was assessed using multivariable logistic regression model adjusting 15 potential confounders.

**Results:**

A total of 69319 patients were included, and 808 died among them. A 100-ml increase in FFP transfusion volume was associated with a higher in-hospital mortality (odds ratio 1.05, 95% confidence interval 1.04–1.06, *p* < 0.001) after controlling for the confounders. FFP transfusion volume was also associated with superficial surgical site infection, nosocomial infection, prolonged length of hospital stay, ventilation time, and acute respiratory distress syndrome. The significant association between FFP transfusion volume and in-hospital mortality was extended to the subgroups of cardiac surgery, vascular surgery, and thoracic or abdominal surgery.

**Conclusions:**

A higher volume of perioperative FFP transfusion was associated with an increased in-hospital mortality and inferior postoperative outcomes in surgical patients without MT.

## 1. Introduction

Massive transfusion (MT) is defined as transfusion of ≥10 units (U) of red blood cell (RBC) within 24 h (1). An early and aggressive plasma transfusion has been found to be associated with a decreased mortality in traumatic patients requiring MT (2–4). Guidelines recommended to deliver plasma in parallel with the first unit of RBC in a predesigned ratio (commonly greater than 1:2) in trauma settings when a MT is anticipated (5); and this strategy has been generalized to non-traumatic surgical bleeding conditions (6, 7). However, it is sometimes difficult to accurately predict whether an active surgical bleeding will develop into massive bleeding (8, 9). Goal-directed coagulation management is also infeasible in centers where point-of-care coagulation monitoring is inaccessible. Thus, the ratio-driven resuscitation may be overactivated in non-massively transfused patients (10), and the effects of plasma transfusion are unclear in these patients. In traumatic patients without MT, a high ratio of plasma to RBC may not improve survival, and can even be associated with increased risks of complications (11, 12). Up to now, there is a paucity of research assessing the survival impact of plasma transfusion in non-massively transfused surgical patients.

With these questions in mind, the primary aim of this study was to investigate the association between the volume of fresh frozen plasma (FFP) transfusion and in-hospital mortality in surgical patients without preoperative coagulopathy and not receiving MT. We hypothesized that a higher volume of FFP transfusion would be associated with an increased risk of in-hospital mortality.

## 2. Materials and methods

The research protocol was approved by the institutional review board (IRB) of Peking Union Medical College Hospital (reference number: S-K1047). The requirement of written informed consent was waived by the IRB, since all the data was recorded anonymously without any information that may help to identify certain participant. The manuscript adhered to the applicable STROBE guidelines.

### 2.1. Study design and data source

We performed a nationwide retrospective cohort study using data from Hospital Quality Monitoring System (HQMS) managed by National Health Commission of China. Launched in 2011, the system was designed to monitor and improve healthcare quality. HQMS collected anonymized inpatient medical records from all the 31 province-level regions in mainland China. By August 2018, the system has already covered 50% of all the tertiary hospitals. The medical records contained demographic information, diagnoses on admission and discharge, discharge status (alive or dead), procedures performed during hospitalization, complications of the procedures, and transfusion information (the volume and types of blood products transfused if any). Diagnoses and procedures were coded according to the 10^th^ revision of International Classification of Diseases (ICD-10) and 9^th^ revision of International Classification of Diseases, Clinical Modification (ICD-9-CM), respectively.

### 2.2. Study population

Based on HQMS, we included the patients who had at least one record of surgical procedure and received RBC transfusion on the day of surgery in tertiary hospitals from January 2016 to August 2018. Our study focused on patients who did not receive MT, which was defined as a transfusion of ≥10U RBC within 24 h (1); thus, we excluded the patients who received ≥10U RBC in total on the day of surgery. We also excluded the patients who were diagnosed with coagulopathy at admission based on the ICD codes of admission diagnoses (Supplementary Table 1).

### 2.3. Definition of variables

The exposure variable was the volume of FFP transfused on the day of surgery. The primary outcome was in-hospital mortality, which was identified by the discharge status in the HQMS.

There were five secondary outcomes: superficial surgical wound infection (SSI), nosocomial infection, length of hospital stay (LOS), ventilation time, and acute respiratory distress syndrome (ARDS). Superficial surgical wound infection was defined as bad wound healing status. The healing status is coded as “good,” “medium,” “bad,” and “unclear” in China (13), and “bad” healing referred to the wounds with any signs of infection or pus. Nosocomial infection was reported by the surgeons and recorded in HQMS. Ventilation time was defined as postoperative ventilation time in intensive care unit, and categorized as 0 h, >0 and ≤24 h, >0 and ≤72 h, and >72 h. ARDS was identified by the ICD codes of discharge diagnoses.

We defined 15 potential confounders, including age, sex, American Society of Anesthesiology Physical Status Classification (ASA) ≥ III, anemia, malignancy, cerebrovascular disease, heart failure, respiratory failure, hepatic failure, renal failure, sepsis, multiple organ dysfunction syndrome (MODS), trauma, emergency, and total units of RBC transfused on the day of surgery. The identification of the comorbidities was based on the ICD codes of admission diagnoses (Supplementary Table 2).

It was mandatory to transfer the data of diagnoses, procedures, discharge status, and transfusion information to HQMS; thus, there was no missing data of these variables. The data of emergency was missing in 54.7% of the included patients. After consulting the administrators of the HQMS, the reason for missing is the incompatible data interface during the data capture process in some eligible hospitals, which may probably happen in a random manner irrelevant to patients’ or hospitals’ characteristics. For the integrity of the analysis, we did not exclude the patients with missing data of emergency. Instead, we categorized the status of this variable into “yes,” “no,” and “missing data.”

### 2.4. Statistical analysis

The distribution of the continuous variables was examined using visual inspection of the histogram. Continuous normally distributed data and categorical data was described as mean ± standard deviation and number (percentage), respectively. Comparisons between the patients receiving and not receiving FFP transfusion were conducted using standardized difference (SD), where a value smaller than 0.1 was considered as acceptable deviation.

The relationship between the volume of FFP transfused and the primary outcome was assessed using multivariable logistic regression model. We built two models. Model 1 adjusted all the potential confounders. In addition to the confounders in model 1, model 2 included the interaction term of FFP and RBC volume, to test whether the effect of FFP transfusion on mortality differed with different units of RBC transfused.

We also assessed the associations between FFP volume and the secondary outcomes (except LOS) in the logistic regression model using the similar confounding adjustment strategy with model 1 in the primary outcome. LOS was analyzed using a Fine and Gray competing risk survival model with outcome being time to discharge alive adjusting the same 15 confounders. In-hospital death was considered as the competing risk of discharge in the analysis.

In the subgroup analysis, we conducted the above regression analysis (model 1) in patients undergoing cardiac, vascular, thoracic/abdominal, and orthopedic surgeries, respectively. Furthermore, we also would like to investigate the association between FFP volume and mortality when different units of RBC were transfused. Therefore, we divided the included patients using 2, 4, 6, and 8 units of RBC as thresholds, and the associations were assessed in each subgroup.

A total of 34,827 patients in FFP transfusion group and 34,492 patients in control group provide 80.9% statistical power to detect a statistically significant odds ratio of 1.3 given a population risk of 0.59% and two-sided α of 0.05. Data cleaning and analysis were completed in R (R Foundation for Statistical Computing, Vienna, Austria, version 3.5.2) and a two-sided *p* value of 0.05 was considered as statistically significant. We did not adjust for the probability of type I errors in secondary outcomes and subgroup analysis; hence, relevant findings were only considered exploratory.

## 3. Results

**FIGURE 1:**
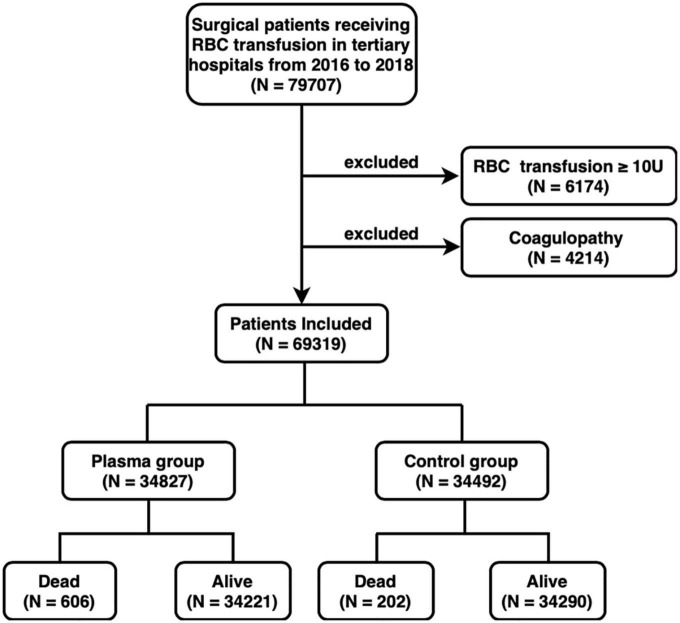
Flowchart of patient selection. RBC, red blood cell; U, unit; FFP, fresh frozen plasma.

There were 79,707 patients receiving at least one surgical procedure and RBC transfusion in tertiary hospitals from 2016 to 2018 (Figure 1). Among them, 6,714 (8.4%) patients were excluded because they received transfusions of ≥10U RBC on the day of surgery. A total of 4,214 (5.3%) patients diagnosed with coagulopathy were also excluded. A total of 69,319 patients were finally included, with 34,827 (50.2%) received FFP transfusion and 34,492 (49.8%) did not. In the FFP group, patients received 400 [200, 900] ml FFP. About 606 (1.7%) and 202 (0.6%) patients dead during hospitalization in the FFP group and the control group, respectively. The alive and dead patients received 0 [0, 400] ml FFP and 400 [0, 1,000] ml FFP, respectively. The three leading surgery types were cardiac, arthroplasty, and abdominal surgeries (Supplementary Table 3).

In the baseline comparison, the FFP group and the control group showed significant differences in age (SD 0.196), sex (SD 0.147), ASA ≥ III (SD 0.118), anemia (SD 0.173), heart failure (SD 0.123), renal failure (SD 0.100), emergency (SD 0.129), and units of RBC transfused (SD 0.458) (Table 1). The information of emergency was missing in 19,869 (57.4%) patients in the FFP group and 18,057 (52.4%) patients in the control group.

**TABLE 1:** Comparison of the baseline data between the FFP group and the control group.

Variables	FFP group (*N* = 34827)^a^	Control group (*N* = 34492)^b^	Standardized difference^c^
Age (year)	53.94 ± 20.87	50.16 ± 21.14	0.196^c^
Male	19,936 (57.2%)	17,840 (51.7%)	0.147^c^
ASA ≥ III	12,462 (35.8%)	10,887 (31.6%)	0.118^c^
Anemia	2,052 (5.9%)	3,565 (10.3%)	0.173^c^
Malignancy	5,610 (16.1%)	5,142 (14.9%)	0.056
Cerebrovascular disease	79 (0.2%)	24 (0.1%)	0.042
Heart failure	1,063 (3.1%)	514 (1.5%)	0.123^c^
Respiratory failure	220 (0.6%)	148 (0.4%)	0.044
Hepatic failure	74 (0.2%)	21 (0.1%)	0.057
Renal failure	332 (1.0%)	690 (2.0%)	0.100^c^
Sepsis	391 (1.1%)	197 (0.6%)	0.077
MODS	79 (0.2%)	24 (0.1%)	0.056
Trauma	5,505 (15.8%)	5,501(15.9%)	0.019
Emergency			0.129^c^
Yes	4,109 (11.8%)	4,307 (12.5%)	
No	10,397 (29.9%)	12,463 (36.1%)	
Missing data	19,869 (57.4%)	18,057 (52.4%)	
Units of RBC	4.0 [2.0, 6.0]	3.0 [2.0, 4.0]	0.458^c^

FFP, fresh frozen plasma; ASA, American Society of Anesthesiology; MODS, multiple organ dysfunction syndrome; RBC, red blood cell.

^a, b^Continuous data were expressed as mean ± standard difference (normally distributed data) or median [interquartile range] (non-normally distributed data), and categorical data were described as number (percentage).

^c^Standardized difference ≥ 0.1.

In the analysis of the primary outcome, a 100-ml increase in FFP transfusion volume was associated with an approximately 5% increased odds of in-hospital mortality [odds ratio (OR) 1.05, 95% confidence interval (CI) 1.04–1.06, *p* < 0.001] after the adjustment of 15 potential confounders (Table 2). When we included the interaction term of FFP and RBC into the multivariable model, the association between the volume of FFP and mortality was still significant (OR 1.07, 95% CI 1.05–1.08, *p* < 0.001) (Table 2). The interaction between FFP and RBC was statistically significant (OR 1.00, 95% CI 0.99–1.00, *p* 0.013). However, we believed the OR did not indicate clinical significance.

**TABLE 2:** Multivariable logistic regression analysis of the in-hospital mortality (*N* = 69319).

Variables	Model 1			Model 2		
	OR	95% CI	*p*	OR	95% CI	*p*
FFP (100 ml)	1.05	1.04–1.06	<0.001	1.07	1.05–1.08	<0.001
Age (10 years)	1.15	1.10–1.20	<0.001	1.15	1.11–1.20	<0.001
Male vs. female	1.34	1.15–1.57	<0.001	1.34	1.15–1.57	<0.001
ASA ≥ III	2.31	1.99–2.69	<0.001	2.31	1.99–2.69	<0.001
Anemia	0.85	0.65–1.10	0.230	0.85	0.65–1.10	0.227
Malignancy	1.25	1.02–1.54	0.031	1.24	1.01–1.52	0.039
Cerebrovascular disease	4.17	2.71–6.15	<0.001	4.18	2.72–6.17	<0.001
Heart failure	1.83	1.31–2.51	<0.001	1.84	1.31–2.52	<0.001
Respiratory failure	18.52	13.87–24.55	<0.001	18.35	13.73–24.32	<0.001
Hepatic failure	5.29	2.51–10.44	<0.001	5.32	2.53–10.49	<0.001
Renal failure	2.06	1.33–3.07	0.001	2.05	1.33–3.06	0.001
Sepsis	1.57	1.05–2.28	0.023	1.54	1.03–2.24	0.030
MODS	35.34	21.67–57.30	<0.001	34.62	21.23–56.18	<0.001
Trauma	1.75	1.45–2.11	<0.001	1.74	1.44–2.10	<0.001
**Emergency**						
Missing data (ref)	1.00	NA		1.00	NA	
Yes	1.52	1.26–1.84	<0.001	1.52	1.26–1.84	<0.001
No	0.69	0.58–0.83	<0.001	0.69	0.57–0.83	<0.001
RBC (U)	1.09	1.06–1.13	<0.001	1.12	1.08–1.16	<0.001
FFP (100ml)*RBC(U)^a^	NA			1.00	0.99–1.00	0.013

FFP, fresh frozen plasma; OR, odds ratio; CI, confidence interval; ASA, American Society of Anesthesiology; MODS, multiple organ dysfunction syndrome; ref, reference; RBC, red blood cell; U, unit; NA, not applicable.

^a^Interaction term of the volume of FFP and the volume of red blood cell.

In the analysis of the secondary outcomes, a 100-ml increase of FFP transfusion volume was significantly associated with superficial SSI (OR 1.03, 95% CI 1.02–1.04, *p* < 0.001), nosocomial infection (OR 1.03, 95% CI 1.02–1.04, *p* < 0.001), LOS [hazard ratio (HR) 1.05, 95% CI 1.04–1.07, *p* < 0.001], ventilation time (OR 1.03, 95% CI 1.03–1.04, *p* < 0.001), and ARDS (OR 1.03, 95% CI 1.00–1.05, *p* 0.016) after controlling for the 15 potential confounders (Table 3).

**TABLE 3:** Analysis of the secondary outcomes.

Secondary outcome	FFP group^a^ (*N* = 34827)	Control group^b^ (*N* = 34492)	FFP (100 ml) in the model^c^		
			OR/HR	95% CI	*p*
Superficial SSI	545 (1.6%)	377 (1.1%)	1.03	1.02–1.04	<0.001
Nosocomial infection	792 (2.3%)	562 (1.6%)	1.03	1.02–1.04	<0.001
LOS (d)	20 [15,26]	21 [16,28]	1.05	1.04–1.07	<0.001
Ventilation time (h)			1.03	1.03–1.04	<0.001
0	18715 (53.7%)	20905 (60.6%)			
>0, ≤24	4441 (12.8%)	2373 (6.9%)			
>0, ≤72	544 (1.6%)	198 (0.6%)			
>72	454 (1.3%)	156 (0.5%)			
ARDS	33 (0.09%)	17 (0.05%)	1.03	1.00–1.05	0.016

SSI, surgical site infection; FFP, fresh frozen plasma; OR, odds ratio; HR, hazard ratio; CI, confidence interval; No., number; ARDS, acute respiratory distress syndrome. LOS, length of hospitalization.

^a,b^Continuous data were expressed as median [interquartile range] (non-normally distributed data), and categorical data were described as number (percentage).

^c^LOS was analyzed using a Fine and Gray competing risk survival model with outcome being time to discharge alive, and in-hospital death was considered as the competing risk of discharge in the time-to-event analysis. The other outcomes were analyzed using multiple regression model. Ventilation time was categorized using 0, 24, and 72 h as thresholds, and analyzed by ordinal logistic regression. Cerebrovascular disease and hepatic failure were excluded from the model of ARDS, since the events were too small. Similarly, hepatic failure and MODS were excluded from the model of ventilation time.

In the subgroup analysis, the significant association between the volume of FFP transfusion and in-hospital mortality was extended to the subgroups of cardiac surgery (OR 1.08, 95% CI 1.04–1.11, *p* < 0.001), vascular surgery (OR 1.05, 95% CI 1.02–1.07, *p* 0.001), and thoracic or abdominal surgery (OR 1.05, 95% CI 1.03–1.06, *p* < 0.001). The volume of FFP transfusion is associated with in-hospital mortality, regardless of the units of RBC transfused, and the OR tended to decrease as the units of RBC increased (>0 and ≤2U: OR 1.06, 95% CI 1.05–1.08, *p* < 0.001; >2 and ≤4U: OR 1.05, 95% CI 1.04–1.07, *p* < 0.001; >4 and ≤6U: OR 1.05, 95% CI 1.03–1.06, *p* < 0.001; >6 and ≤8U: OR 1.04, 95% CI 1.01–1.06, *p* 0.001; >8 and ≤10U: OR 1.04, 95% CI 1.02–1.06, *p* < 0.001) (Table 4).

**TABLE 4:** Multivariable logistic regression analysis of in-hospital mortality in subgroups.

Subgroup	Total No.	No.(%) of in-hospital Mortality		FFP (100 ml) in multiple regression model^a^		
		FFP group	Control group	OR	95% CI	*p*
**Surgery type**						
Cardiac^b^	8501	66 (1.0%)	5 (0.3%)	1.08	1.04–1.11	<0.001
Vascular	2177	51 (4.6%)	16 (1.5%)	1.05	1.02–1.07	0.001
Thoracic or abdominal	11464	148 (2.2%)	42 (0.9%)	1.05	1.03–1.06	<0.001
Orthopedic	11928	15 (0.4%)	9 (0.1%)	1.08	0.98–1.15	0.065
**Units of RBC**						
0 < RBC ≤ 2	26550	125 (1.2%)	87 (0.5%)	1.06	1.05–1.08	<0.001
2 < RBC ≤ 4	24952	183 (1.5%)	68 (0.5%)	1.05	1.04–1.07	<0.001
4 < RBC ≤ 6	9654	124 (2.1%)	27 (0.7%)	1.05	1.03–1.06	<0.001
6 < RBC ≤ 8	5207	92 (2.6%)	16 (1.0%)	1.04	1.01–1.06	0.001
8 < RBC ≤ 10	2956	82 (3.5%)	4 (0.6%)	1.04	1.02–1.06	<0.001

FFP, fresh frozen plasma; OR, odds ratio; CI, confidence interval; No., number; RBC, red blood cell.

^a^The event number of malignancy, respiratory failure, sepsis, multiple organ dysfunction syndrome (MODS), and emergency were too small, thus they were excluded from the regression model in cardiac subgroup. Similarly, malignancy, respiratory failure, sepsis, and hepatic failure were excluded from the model in vascular subgroup; malignancy, Cerebrovascular disease, respiratory failure, heart failure, hepatic failure, renal failure, sepsis, and MODS were excluded from the model in vascular subgroup; MODS were excluded from 8 < RBC ≤ 10 group.

^b^In the cardiac subgroup, age was analyzed by restricted cubic splines due to the non-linear relationship between age and mortality.

## 4. Discussion

In this nationwide cohort study, after the adjustment of 15 confounders, we found that a higher volume of perioperative FFP transfusion was associated with increased risks of in-hospital mortality and postoperative complications in patients undergoing surgery without MT.

Our study raised a question why FFP transfusion was administrated in 50.2% of the included patients. Since we cannot trace back the clinical scenario of each case in this retrospective study, we inferred two possible reasons. First, some of the included patients might initially be transfused with a fixed ratio of FFP and RBC because a MT was anticipated. However, the acute intraoperative bleeding was rapidly arrested; thus, they did not receive ≥10U RBC in one episode. To the best of our knowledge, there is scare evidence on the trigger of this ratio-driven strategy in surgical bleeding. Considering the associated risks, physicians should terminate aggressive FFP transfusion immediately when they recognize MT is not required. The second reason is the inability to utilize point-of-care coagulation tests. A survey in the US indicated that only 9% of the institutions routinely perform thromboelastography or rotational thromboelastometry in MT (14), and the situation may be even worse in China. Without the point-of-care data, physicians sometimes initiate FFP transfusion when they observe extensive oozing in surgical fields; thus, the transfusion decision is affected by their personal experiences and preferences (15). This may lead to inappropriate indication for FFP transfusion and inadequate transfusion dose. In our study, the dead patients received 400 [0, 1,000] ml FFP, a dose far less than the recommended 10 ml/kg, which could not achieve a clinically significant increase in coagulation factors (16).

In our study, the exposure variable was the volume of FFP transfusion, rather than the ratio of FFP to RBC like in previous studies (2–4, 17–20). Since RBC transfusion was observed to be associated with mortality in surgical patients (21, 22), this factor should be well controlled as an essential confounder in the data analysis. If the ratio of FFP and RBC was used as the exposure in the regression model, including the RBC transfusion again in the model as a confounder would lead to severe problem of collinearity due to the mathematically negative association between the ratio and RBC transfusion, which might distort estimate of the exposure. Hence, we did not choose the ratio as the exposure variable.

Several observational studies found a high FFP to RBC ratio was associated with decreased 24-hour and 30-day mortality in severe traumatic patients (2–4, 23), while a randomized controlled trial detected no significant difference of mortality (24). Our findings are inconsistent with these results possibly for the following two reasons. First, one third of the traumatic patients present early coagulopathy at hospital admission (25), which is induced by clotting factor consumption, hypoperfusion, acidosis, and hypothermia (26). These dysfunctions can be reversed by a balanced transfusion of plasma and RBC (27). On the contrary, we excluded the patients with preoperative coagulopathy or receiving MT in this study. Furthermore, for surgical patients, acute hemorrhage was most likely to occur during the surgery, the intraoperative close monitoring allows physicians to timely detect and control hemorrhage. Therefore, the included patients were less likely to develop coagulopathy, and might not benefit from plasma transfusion. Second, compared with traumatic patients, surgical patients tend to be older and with higher burdens of comorbidities (17, 28), which can diminish the survival benefits of plasma transfusion.

The effects of FFP transfusion were controversial in non-traumatic surgical patients. Some studies focused on cardiac surgery indicated that a high FFP-to-RBC ratio in MT can improve survival (18, 19, 29). In contrast, according to the observation in a large tertiary hospital, a greater intraoperative plasma transfusion volume was associated with fewer hospital-free days in heterogenous surgeries with or without MT (17, 30, 31). Multicenter cohort studies also suggested a significant association between FFP transfusion and increased mortality in patients with gastrointestinal bleeding (32–34). Previous studies mostly focused on cardiac, vascular, and transplant surgeries (17–20, 29–31, 35, 36), while our study additionally provided evidence for a wide range of surgeries. In the subgroup analysis, the association between plasma transfusion volume and mortality was significant in cardiac, vascular and thoracic/abdominal surgeries, but not in orthopedic surgery. Orthopedic surgery is associated with a large amount of hidden blood loss, which is very likely to be underestimated in clinical practice (37). Therefore, some included orthopedic patients might actually suffer from greater blood loss and should receive MT. This may explain the insignificant result of orthopedic surgery.

Since the survival benefits of FFP transfusion have been demonstrated in MT (2–4, 20, 29), we explored whether effects of FFP volume changed as the units of RBC transfused increased within the range of 0-10U. However, we did not find any relevant evidence. In the multivariable regression model 2, the association of FFP volume on mortality did not clinically significantly differ with the RBC units (Table 2). Similarly, in the subgroup analysis, the association between FFP volume and mortality was significant no matter how much RBC transfused (Table 4).

In addition to in-hospital mortality, we also found that FFP volume was significantly associated with inferior postoperative outcomes, including superficial SSI, nosocomial infection, LOS, ventilation time, and ARDS. The association between FFP transfusion and postoperative infection has also been reported in patients undergoing cardiac and esophageal surgery (22, 38). The potential mechanism may be the immunomodulatory effect of FFP on monocyte that has been observed in preclinical studies (39, 40). The presence of donors’ extracellular DNA in FFP may also play a significant role. Extracellular DNA can be transferred to recipients during FFP transfusion, where it can activate innate immune system and triggers inflammatory responses (41). Furthermore, FFP transfusion may increase the risk of postoperative pulmonary complications. Transfusion-related lung injury (TRALI) and circulatory overload are both adverse effects of FFP transfusion, and can lead to prolonged mechanical ventilation time in the aftermath of transfusion (42). Particularly, TRALI commonly presents as the onset of ARDS following transfusion (43).

The limitations of our study lie in the following aspects. First, as an observational study, our findings cannot imply causality. Second, despite our attempts to adjust the confounding effects of 15 comorbidities, there are some other potential confounders that were not recorded in HQMS, such as body mass index, operation duration and difficulty, estimated blood loss, the amount and type of fluid infused intraoperatively, and the use of anticoagulant, antiplatelet, or prohemostatic agents. Therefore, the significant association between FFP transfusion and mortality might be attributed to these unadjusted confounders. Third, we identified the confounders using ICD codes of diagnoses, which were highly depended on physician’s judgments in a real-world practice; thus, misclassification bias could not be excluded. However, since the data was collected from tertiary hospitals where physicians were well trained, we believed that the diagnoses were basically reliable. Finally, FFP transfusion was found to be associated with MODS and respiratory failure (44). Thus, bias might be introduced when we adjusted the confounding effects of these two potentially intermediate variables. However, this should be a protective bias that weakened the associations between FFP volume and the outcomes. Since the associations were still significant after this overadjustment, we believed the actual associations should be stronger and robust.

Based on our findings, physicians should cautiously assess the indications for FFP transfusion and the associated risks in surgical patients without MT. Special attentions should be paid to patients receiving high volumes of FFP.

## Data availability statement

The raw data supporting the conclusions of this article will be made available from by the authors on reasonable request.

## Ethics statement

The studies involving human participants were reviewed and approved by Institutional Review Board of Peking Union Medical College Hospital. The ethics committee waived the requirement of written informed consent for participation.

## Author contributions

XX, YZ, and XY: study design. XX and YZ: data acquisition. XX, YZ, and BT: data analysis/interpretation and drafting of manuscript. XY and YH: critical revision of manuscript. All authors agreed to be accountable for the content of the work.

## References

[1] Pham H Shaz B. Update on massive transfusion. *Br J Anaesth.* (2013) 111(Suppl. 1):i71–82. 10.1093/bja/aet376 24335401

[2] Roquet F Neuschwander A Hamada S Fave G Follin A Marrache D Association of early, high plasma-to-red blood cell transfusion ratio with mortality in adults with severe bleeding after trauma. *JAMA Netw Open.* (2019) 2:e1912076. 10.1001/jamanetworkopen.2019.12076 31553473 PMC6763975

[3] Bui E Inaba K Ebadat A Karamanos E Byerly S Okoye O The impact of increased plasma ratios in massively transfused trauma patients: a prospective analysis. *Eur J Trauma Emerg Surg.* (2016) 42:519–25. 10.1007/s00068-015-0573-1 26362535

[4] Bhangu A Nepogodiev D Doughty H Bowley D. Meta-analysis of plasma to red blood cell ratios and mortality in massive blood transfusions for trauma. *Injury.* (2013) 44:1693–9. 10.1016/j.injury.2012.07.193 23021369

[5] Jones A Frazier S. Association of blood component ratio with clinical outcomes in patients after trauma and massive transfusion: a systematic review. *Adv Emerg Nurs J.* (2016) 38:157–68. 10.1097/TME.0000000000000103 27139137

[6] Baumann Kreuziger L Morton C Subramanian A Anderson C Dries D. Not only in trauma patients: hospital-wide implementation of a massive transfusion protocol. *Transfus Med.* (2014) 24:162–8. 10.1111/tme.12096 24372790 PMC4043857

[7] Patel E Ness P Marshall C Gniadek T Efron D Miller P Blood product utilization among trauma and nontrauma massive transfusion protocols at an urban academic medical center. *Anesth Analg.* (2017) 125:967–74. 10.1213/ANE.0000000000002253 28719428

[8] Callcut R Cotton B Muskat P Fox E Wade C Holcomb J Defining when to initiate massive transfusion: a validation study of individual massive transfusion triggers in PROMMTT patients. *J Trauma Acute Care Surg.* (2013) 74:59–65, 67–8; discussion66–7. 10.1097/TA.0b013e3182788b34 23271078 PMC3771339

[9] Foster J Sappenfield J Smith R Kiley S. Initiation and termination of massive transfusion protocols: current strategies and future prospects. *Anesth Analg.* (2017) 125:2045–55. 10.1213/ANE.0000000000002436 28857793

[10] Bell C Prokopchuk-Gauk O Cload B Stirling A Davis P. Optimum accuracy of massive transfusion protocol activation: the clinician’s view. *Cureus.* (2018) 10:e3688. 10.7759/cureus.3688 30761240 PMC6368427

[11] Inaba K Branco B Rhee P Blackbourne L Holcomb J Teixeira P Impact of plasma transfusion in trauma patients who do not require massive transfusion. *J Am Coll Surg.* (2010) 210:957–65. 10.1016/j.jamcollsurg.2010.01.031 20510805

[12] Sambasivan C Kunio N Nair P Zink K Michalek J Holcomb J High ratios of plasma and platelets to packed red blood cells do not affect mortality in nonmassively transfused patients. *J Trauma.* (2011) 71:S329–36. 10.1097/TA.0b013e318227edd3 21814100

[13] Zhang Y. Surgical wound classification on home page of medical records and definition standard of healing grade. *Chin Med Record.* (2006) 7:22.

[14] Etchill E Sperry J Zuckerbraun B Alarcon L Brown J Schuster K The confusion continues: results from an American association for the surgery of trauma survey on massive transfusion practices among United States trauma centers. *Transfusion.* (2016) 56:2478–86. 10.1111/trf.13755 27515056

[15] Unal D Senayli Y Polat R Spahn D Toraman F Alkis N Peri-operative blood transfusion in elective major surgery: incidence, indications and outcome–an observational multicentre study. *Blood Transfus.* (2020) 18:261–79. 32697928 10.2450/2020.0011-20 PMC7375885

[16] Stanworth S Grant-Casey J Lowe D Laffan M New H Murphy M The use of fresh-frozen plasma in England: high levels of inappropriate use in adults and children. *Transfusion.* (2011) 51:62–70. 10.1111/j.1537-2995.2010.02798.x 20804532

[17] Warner M Frank R Weister T Madde N Gajic O Kor D. Ratios of plasma and platelets to red blood cells in surgical patients with acute intraoperative hemorrhage. *Anesth Analg.* (2020) 131:483–93. 10.1213/ANE.0000000000004609 31880628 PMC7580504

[18] Tsukinaga A Maeda T Takaki S Michihata N Ohnishi Y Goto T. Relationship between fresh frozen plasma to packed red blood cell transfusion ratio and mortality in cardiovascular surgery. *J Anesth.* (2018) 32:539–46. 10.1007/s00540-018-2508-6 29789931

[19] Delaney M Stark P Suh M Triulzi D Hess J Steiner M Massive transfusion in cardiac surgery: the impact of blood component ratios on clinical outcomes and survival. *Anesth Analg.* (2017) 124:1777–82. 10.1213/ANE.0000000000001926 28333704 PMC5438286

[20] Teixeira P Inaba K Karamanos E Rhee P Shulman I Skiada D The survival impact of plasma to red blood cell ratio in massively transfused non-trauma patients. *Eur J Trauma Emerg Surg.* (2017) 43:393–8. 10.1007/s00068-016-0674-5 27117790

[21] Vlot E Verwijmeren L van de Garde E Kloppenburg G van Dongen E Noordzij P. Intra-operative red blood cell transfusion and mortality after cardiac surgery. *BMC Anesthesiol.* (2019) 19:65. 10.1186/s12871-019-0738-2 31054585 PMC6499947

[22] Ming Y Liu J Zhang F Chen C Zhou L Du L Transfusion of red blood cells, fresh frozen plasma, or platelets is associated with mortality and infection after cardiac surgery in a dose-dependent manner. *Anesth Analg.* (2020) 130:488–97. 10.1213/ANE.0000000000004528 31702696

[23] da Luz L Shah P Strauss R Mohammed A D’Empaire P Tien H Does the evidence support the importance of high transfusion ratios of plasma and platelets to red blood cells in improving outcomes in severely injured patients: a systematic review and meta-analyses. *Transfusion.* (2019) 59:3337–49. 10.1111/trf.15540 31614006 PMC6900194

[24] Holcomb J Tilley B Baraniuk S Fox E Wade C Podbielski J Transfusion of plasma, platelets, and red blood cells in a 1:1:1 vs a 1:1:2 ratio and mortality in patients with severe trauma: the PROPPR randomized clinical trial. *JAMA.* (2015) 313:471–82. 25647203 10.1001/jama.2015.12 PMC4374744

[25] Spahn D Bouillon B Cerny V Duranteau J Filipescu D Hunt B The European guideline on management of major bleeding and coagulopathy following trauma: fifth edition. *Crit Care.* (2019) 23:98. 10.1186/s13054-019-2347-3 30917843 PMC6436241

[26] Chang R Cardenas J Wade C Holcomb J. Advances in the understanding of trauma-induced coagulopathy. *Blood.* (2016) 128:1043–9. 10.1182/blood-2016-01-636423 27381903 PMC5000842

[27] Holcomb J Jenkins D Rhee P Johannigman J Mahoney P Mehta S Damage control resuscitation: directly addressing the early coagulopathy of trauma. *J Trauma.* (2007) 62:307–10. 10.1097/TA.0b013e3180324124 17297317

[28] Holcomb J del Junco D Fox E Wade C Cohen M Schreiber M The prospective, observational, multicenter, major trauma transfusion (PROMMTT) study: comparative effectiveness of a time-varying treatment with competing risks. *JAMA Surg.* (2013) 148:127–36. 10.1001/2013.jamasurg.387 23560283 PMC3740072

[29] Mazzeffi M Chriss E Davis K Zhan M Harris A Rock P Optimal plasma transfusion in patients undergoing cardiac operations with massive transfusion. *Ann Thorac Surg.* (2017) 104:153–60. 10.1016/j.athoracsur.2016.09.071 27964918

[30] Warner M Frank R Weister T Smith M Stubbs J Kor D. Higher intraoperative plasma transfusion volumes are associated with inferior perioperative outcomes. *Transfusion.* (2019) 59:112–24. 10.1111/trf.14988 30383908 PMC6322936

[31] Smith M Kor D Frank R Weister T Dearani J Warner M. Intraoperative plasma transfusion volumes and outcomes in cardiac surgery. *J Cardiothorac Vasc Anesth.* (2020) 34:1446–56. 10.1053/j.jvca.2019.12.049 32044241 PMC7198357

[32] Mohanty A Kapuria D Canakis A Lin H Amat M Rangel Paniz G Fresh frozen plasma transfusion in acute variceal haemorrhage: results from a multicentre cohort study. *Liver Int.* (2021) 41:1901–8. 10.1111/liv.14936 33969607

[33] Liu S Zhang X Walline J Yu X Zhu H. Fresh frozen plasma in cases of acute upper gastrointestinal bleeding does not improve outcomes. *Front Med (Lausanne).* (2022) 9:934024. 10.3389/fmed.2022.934024 35911402 PMC9330331

[34] Subramaniam K Spilsbury K Ayonrinde O Latchmiah F Mukhtar S Semmens J Red blood cell transfusion is associated with further bleeding and fresh-frozen plasma with mortality in nonvariceal upper gastrointestinal bleeding. *Transfusion.* (2016) 56:816–26. 10.1111/trf.13446 26718025

[35] Saxena A Chua T Fransi S Liauw W Morris D. Effectiveness of early and aggressive administration of fresh frozen plasma to reduce massive blood transfusion during cytoreductive surgery. *J Gastrointest Oncol.* (2013) 4:30–9. 23449950 10.3978/j.issn.2078-6891.2012.046 PMC3562619

[36] Phillips A Tran L Foust J Liang N. Systematic review of plasma/packed red blood cell ratio on survival in ruptured abdominal aortic aneurysms. *J Vasc Surg.* (2021) 73:1438–44. 10.1016/j.jvs.2020.10.027 33189763 PMC8005448

[37] Good L Peterson E Lisander B. Tranexamic acid decreases external blood loss but not hidden blood loss in total knee replacement. *Br J Anaesth.* (2003) 90:596–9. 10.1093/bja/aeg111 12697586

[38] Subramanian A Berbari E Brown M Allen M Alsara A Kor D. Plasma transfusion is associated with postoperative infectious complications following esophageal resection surgery: a retrospective cohort study. *J Cardiothorac Vasc Anesth.* (2012) 26:569–74. 10.1053/j.jvca.2011.12.015 22336690

[39] Shah S Coppolino K Menocha S Beceiro S Nateri J Spinella P Immunomodulatory effects of plasma products on monocyte function in vitro. *J Trauma Acute Care Surg.* (2018) 84:S47–53. 10.1097/TA.0000000000001820 29401191

[40] Patlan M Sanchez-Munoz F Amezcua-Guerra L Granados A Paez A Masso F Effect of fresh frozen plasma on the in vitro activation of U937 monocytes: a potential role for the age of blood donors and their underlying cytokine profile. *Biol Res.* (2017) 50:42. 10.1186/s40659-017-0146-3 29268779 PMC5740577

[41] Yang L Yang D Yang Q Cheng F Huang Y. Extracellular DNA in blood products and its potential effects on transfusion. *Biosci Rep.* (2020) 40:BSR20192770. 10.1042/BSR20192770 32150264 PMC7098128

[42] Clifford L Jia Q Subramanian A Yadav H Schroeder D Kor D. Risk factors and clinical outcomes associated with perioperative transfusion-associated circulatory overload. *Anesthesiology.* (2017) 126:409–18. 10.1097/ALN.0000000000001506 28072601 PMC5309147

[43] McVey M Kapur R Cserti-Gazdewich C Semple J Karkouti K Kuebler W. Transfusion-related acute lung injury in the perioperative patient. *Anesthesiology.* (2019) 131:693–715. 10.1097/ALN.0000000000002687 31408449

[44] Watson G Sperry J Rosengart M Minei J Harbrecht B Moore E Fresh frozen plasma is independently associated with a higher risk of multiple organ failure and acute respiratory distress syndrome. *J Trauma.* (2009) 67:221–7; discussion228–30. 19667872 10.1097/TA.0b013e3181ad5957

