# Supplementary material for: Association between perioperative plasma transfusion and in-hospital mortality in patients undergoing surgeries without massive transfusion: A nationwide retrospective cohort study
Source: Front Med (Lausanne). 2023 Feb 15;10:1130359. doi: 10.3389/fmed.2023.1130359 (PMC9975265; doi:10.3389/fmed.2023.1130359)
Supplement: Supplementary file 1 [file Table_1.DOCX]

Supplementary Material

Association between Perioperative Plasma Transfusion and In-hospital Mortality in Patients Undergoing Surgeries without Massive Transfusion: a Nationwide Retrospective Cohort Study

Xiaohan Xu, Yuelun Zhang, Bo Tang, Xuerong Yu, Yuguang Huang*

*** Correspondence:** Yuguang Huang, garypumch@163.com

# Supplementary Table 1 International Classification of Diseases Codes of Coagulopathy

| **ICD-10** | **Description** |
| --- | --- |
| D65 | Disseminated intravascular coagulation |
| D66 | Hereditary factor VIII deficiency |
| D67 | Hereditary factor IX deficiency |
| D68 | Other coagulation defects |
| D68.0 | Von Willebrand's disease |
| D68.1 | Hereditary factor XI deficiency |
| D68.2 | Hereditary deficiency of other clotting factors |
| D68.3 | Hemorrhagic disorder due to circulating anticoagulants |
| D68.31 | Hemorrhagic disorder due to intrns circ anticoag, antib, inhib |
| D68.311 | Acquired hemophilia |
| D68.312 | Antiphospholipid antibody with hemorrhagic disorder |
| D68.318 | Oth hemorrhagic disord d/t intrns circ anticoag, antib, inhib |
| D68.32 | Hemorrhagic disord d/t extrinsic circulating anticoagulants |
| D68.4 | Acquired coagulation factor deficiency |
| D68.8 | Other specified coagulation defects |
| D68.9 | Coagulation defect, unspecified |
| D69 | Purpura and other hemorrhagic conditions |
| O46.0 | Antepartum hemorrhage with coagulation defect |
| O46.00 | Antepartum hemorrhage with coagulation defect, unspecified |
| O46.001 | Antepartum hemorrhage w coag defect, unsp, first trimester |
| O46.002 | Antepartum hemorrhage w coag defect, unsp, second trimester |
| O46.003 | Antepartum hemorrhage w coag defect, unsp, third trimester |
| O46.009 | Antepartum hemorrhage w coag defect, unsp, unsp trimester |
| O46.01 | Antepartum hemorrhage with afibrinogenemia |
| O46.011 | Antepartum hemorrhage with afibrinogenemia, first trimester |
| O46.012 | Antepartum hemorrhage with afibrinogenemia, second trimester |
| O46.013 | Antepartum hemorrhage with afibrinogenemia, third trimester |
| O46.019 | Antepartum hemorrhage with afibrinogenemia, unsp trimester |
| O46.02 | Antepartum hemorrhage w disseminated intravasc coagulation |
| O46.021 | Antepart hemorrhage w dissem intravasc coag, first trimester |
| O46.022 | Antepart hemor w dissem intravasc coag, second trimester |
| O46.023 | Antepart hemorrhage w dissem intravasc coag, third trimester |
| O46.029 | Antepart hemorrhage w dissem intravasc coag, unsp trimester |
| O46.09 | Antepartum hemorrhage with other coagulation defect |
| O46.091 | Antepartum hemorrhage w oth coag defect, first trimester |
| O46.092 | Antepartum hemorrhage w oth coag defect, second trimester |
| O46.093 | Antepartum hemorrhage w oth coag defect, third trimester |
| O46.099 | Antepartum hemorrhage w oth coag defect, unsp trimester |

# Supplementary Table 2 International Classification of Diseases Codes of Confounders

Supplementary Table 2 is too large to be displayed in this Word file. Please see Supplementary Table 2 in the Supplementary excel file.

# Supplementary Table 3 Surgery Types

| **Surgery Type** | **Number (percentage)** |
| --- | --- |
| Cardiac surgery | 8501 (12.3%) |
| Arthroplasty | 8022 (11.6%) |
| Abdominal surgery | 7281 (10.5%) |
| Thoracic surgery | 4183 (6.0%) |
| Spine surgery | 3132 (4.5%) |
| Vascular surgery | 2177 (3.1%) |
| Obstetric surgery | 1012 (1.5%) |
| Neurosurgery | 842 (1.2%) |
| Others | 44558 (49.3%) |
